# Modulation and Apoptosis of Neutrophil Granulocytes by Extracorporeal Photopheresis in the Treatment of Chronic Graft-Versus-Host Disease

**DOI:** 10.1371/journal.pone.0134518

**Published:** 2015-08-04

**Authors:** Cindy Franklin, Elvir Cesko, Uwe Hillen, Bastian Schilling, Sven Brandau

**Affiliations:** 1 Department of Dermatology, Venereology and Allergology, University Hospital Essen, Essen, Germany; 2 Research Division, Department of Otorhinolaryngology, University Hospital Essen, Essen, Germany; University of Tübingen, GERMANY

## Abstract

Chronic graft-versus-host disease (cGVHD) is a common side effect of allogeneic stem cell transplantation and a major cause of morbidity and mortality in affected patients. Especially skin, eyes and oral mucosa are affected. This can lead to pain and functional impairment. Extracorporeal photopheresis (ECP) is an effective immunomodulatory therapy with minimal side effects but its mode of action is still largely unknown. The objective of the present study was to examine the effects of ECP on neutrophil granulocytes in patients with cGVHD. Analysis of leukocytes from cGVHD patients obtained from the ECP device during treatment showed that neutrophil granulocytes account for the majority of cells treated during ECP. Neutrophils from healthy donors treated *in vitro* with 8-methoxypsoralen and UVA light as well as neutrophils from buffy coats of patients with cGVHD treated by ECP showed increased apoptosis and decreased half-life. In remaining non-apoptotic cells chemoirradiation resulted in loss of activation markers and reduced effector functions. This was accompanied by an increase in extracellular arginase-1 activity. Additional comparison of neutrophils isolated from blood of cGVHD patients before and 24h after ECP revealed a decreased half-life and reduction of effector functions of post-ECP neutrophils *ex vivo*. These observations strongly suggest that ECP induces both apoptosis and physiological changes in neutrophils and that these changes also take place *in vivo*. This study is the first to show that ECP modulates apoptosis and inflammatory activity in neutrophil granulocytes, indicating that neutrophils may significantly contribute to the overall immunomodulatory effects attributed to this treatment.

## Introduction

Hematopoietic stem cell transplantation (HSCT) is a curative treatment for hematological malignancies. A large number of patients who have undergone HSCT develop chronic graft-versus-host-disease (cGVHD) [1]. Besides relapse of the malignant disease itself GVHD is the most common cause of death for patients who have undergone stem cell transplantation [2]. Chronic GVHD can affect any organ system, especially skin, eyes and oral mucosa [3]. Skin manifestations include xerosis, discoloration, erosions, ulcerations and sclerosis of skin and subcutaneous fat which cause pain and functional impairment with a major impact on the quality of life [4, 5]. First-line treatment of GVHD consists of immunosuppressive drugs like corticosteroids with limited effectiveness and infections and secondary malignancies as severe side effects [6–8]. Extracorporeal photopheresis (ECP) is an immunomodulatory procedure with few side-effects currently considered standard treatment of steroid-refractory GVHD [9]. ECP treatment can reduce the symptoms of cGVHD and can reduce the immunosuppressive drugs needed for disease control [10, 11]. In several retrospective studies, ECP was applied to heterogeneous patient populations with high response rates [10–14]. It is also applied in a variety of other clinical settings as e.g. for the treatment of cutaneous T cell-lymphoma [15], allograft rejection after solid organ transplantation and severe autoimmune diseases like scleroderma [16]. During ECP, leukocytes are apheresed from venous blood and passage through a plastic chamber, where they are exposed to the photosensitizing drug 8-methoxypsoralen (8-MOP) and then irradiated by UVA light [17]. The modes of action of ECP in GVHD treatment have been studied but still remain largely elusive [18]. Proposed mechanisms include induction of apoptosis in alloreactive lymphocytes [19], induction of regulatory T cells [20–22] and induction of tolerogenic dendritic cells (DC) [19, 23, 24] along with a shift from pro-inflammatory to anti-inflammatory cytokines [25–27]. So far, research was focused on the effects of ECP on mononuclear cells and the role of granulocytes in the treatment of cGVHD by ECP has been largely neglected. It has been shown that ECP leads to distinct homing of neutrophils and mononuclear cells (MNC) [28]. Trautinger et al. showed that ECP can reduce production of respiratory oxygen species [29]. In a recent study, an accumulation of neutrophilic myeloid-derived suppressor cells over time has been observed in a subgroup of patients with GVHD treated with ECP [30]. Here, we demonstrate that neutrophil granulocytes account for the majority of cells treated during ECP and address systematically the effects of this treatment on neutrophil apoptosis and function.

## Materials and Methods

### Patient samples and samples from healthy donors

Peripheral venous blood was taken from healthy donors for in vitro experiments and neutrophil granulocytes isolated as described below. For treatment with 8-methoxypsoralen (8-MOP, UVADEX) and UVA neutrophils were divided into 4 groups: 1.) untreated neutrophils, 2.) neutrophils+ 8-MOP, 3.) neutrophils+UVA light, 4.) neutrophils+8-MOP and UVA light. 340ng/ml 8-MOP was added to group 2 and 4. Group 3 and 4 were irradiated with 2J/cm^2^ using a Bio-Link (Biometra, Göttingen, Germany). Neutrophil granulocytes treated with 8-MOP and UVA are also referred to as chemoirradiated neutrophils throughout the manuscript.

Samples from the Therakos UVAR XTS system (Therakos, USA) were taken from the buffy coat of 16 patients with cGVHD after addition of 8-MOP and irradiation with UVA prior to reinfusion to analyze leukocyte composition. Functional analyses were performed with samples of 4 buffy coats after addition of 8-MOP before and immediately after irradiation with UVA. In addition, peripheral venous blood was taken from 4 patients prior to ECP treatment and 24h later. All patients had received allogeneic hematopoietic stem cell transplantation as a treatment for an underlying hematological disease. Chronic GVHD was scored according to NIH standard by scoring the organ involvement and global severity grading [31]. All human samples were taken after informed written consent and approval by the ethics committee of the medical faculty of the University of Duisburg-Essen.

### Isolation and culture of mononuclear cells and neutrophil granulocytes

Neutrophils, mononuclear cells (MNC) and low-density neutrophils were isolated from blood and buffy coats via density gradient centrifugation as previously described [32, 33]. MNC and neutrophils were resuspended in RPMI-1640 (invitrogen, Karlsruhe, Germany) supplemented with 10% fetal calf serum (Biochrom, Berlin, Germany), 100units/ml penicillin and 100g/ml streptomycin (invitrogen) for functional assays. GCSF was added to all samples to prevent spontaneous apoptosis of neutrophils *ex vivo*.

### Flow cytometric analysis of surface antigens

Antibodies CD11b V450, CD16 PE-Cy7, CD18 PE, CD32PerCP-efluor 710, CD54 APC and CD64 APC-H7 were purchased from BD Bioscience (Heidelberg, Germany) and eBioscience (Frankfurt, Germany). As lineage markers, CD66b-FITC (granulocytes) from Beckman Coulter (Krefeld, Germany), CD3-eFluor 450 (T cells) from eBioscience, CD14-APC-Cy7 (monocytes) from BD Bioscience and CD56-BV510 (NK cells) from BioLegend (Fell, Germany) were used. Appropriate isotype antibodies from eBioscience, BD Bioscience and BioLegend were used as controls (see also S1 Table). Cells were incubated with antibodies for 30 min at 4°C and acquired on a BD FACS Canto II flow cytometer. Analysis and calculations were performed with BD FACS DIVA software.

### Apoptosis staining

MNC and neutrophils were stained with PE-conjugated Annexin V- and 7-AAD (7-amino-actinomycin D) according to the manufacturer’s instructions (BDPharmingen, Heidelberg, Germany) and analyzed by flow cytometry.

### Induced release of reactive oxygen and nitrogen species

Production of reactive nitrogen species (RNS) by neutrophils was determined as described by the manufacturer of DAF-FM diacetate (invitrogen). Quantification of reactive oxygen species (ROS) was determined with Dihydrorhodamine 123 (invitrogen) and measured via flow cytometry according to manufacturer’s instructions.

Briefly: For measurement of reactive nitrogen species, 50 nM PMA (Sigma-Aldrich, Taufkirchen, Germany) and 5 μM DAF-FM diacetate were added to 2x10^5^ neutrophils / well and cultured at 37°C for 45 minutes. Cells were washed with PBS and incubated for additional 15 minutes at 37°C before analysis by flow cytometry.

For quantification of reactive oxygen species, 2x10^5^ cells were stimulated with 50nM PMA after a recovery time of 45min and 30 minutes later Dihydrorhodamine 123 was added at a final concentration of 2.5 μg/ml. Neutrophils were incubated for 15 minutes with Dihydrorhodamine 123 at 37°C and for 15 minutes on ice before measuring with a flow cytometer.

### Generation of supernatants

Neutrophils were resuspended in complete RPMI1640 medium supplemented as above at a concentration of 1x10^6^ cells/ml and cultured for 24h at 37°C with or without LPS (salmonella Minnesota R595, InvivoGen, Toulouse, France,1μg/ml).

### Quantification of cytokines/chemokines

Levels of CXCL8 and CCL4 secreted by neutrophils were analyzed by ELISA according to the protocols provided by the manufacturer (R&D Systems, Wiesbaden, Germany). A Synergy 2 microplate reader (BioTek, Bad Friedrichshall, Germany) was used to determine sample absorbance at 450 nM.

### Quantification of Arginase Activity

Arginase-1 activity in neutrophil supernatants was determined using an Arginase Assay Kit (Abnova, Heidelberg, Germany) according to the manufacturer’s instructions.

Arginase activity was calculated by this formula:

Arginase activity = (ODSample—ODBlank) / (ODStandard—ODWater)*10.4 U/L, where OD is the optical density of the respective samples.

Unit definition: 1 unit of arginase converts 1μmol of L-arginine to ornithine and urea per minute at 37°C and pH 9.5. Sample absorbance was detected in a Synergy 2 microplate reader at 430nM.

### Lymphocyte proliferation

Monocyte-depleted MNC from blood of cGVHD patients 24h after ECP were labeled with proliferation dye efluor 450 (eBioscience). Wells of a 96-well round bottom plate were coated with 100μl 1μg/ml murine IgG2a anti-CD3 (eBioscience) and 1μg/ml murine IgG1 anti-CD28 (Beckmann coulter) in PBS for 2h. 0,05x10^6^ MNC were added per well. Neutrophils from blood of cGVHD patients 24h after ECP were isolated and added at different ratios (MNC: neutrophil ratios: 2.5:1, 5:1, 10:1, 20:1) to autologous and heterologous MNCs, respectively. Cells were left to proliferate for 5 days. Proliferation was measured via flow cytometry after gating on the lymphocyte fraction.

### Statistical Analysis

Statistical analysis was done by Graph Pad Prism 5.0 (Graph Pad Software, La Jolla, CA, USA). Differences were calculated by paired Student’s t-test and One-way- ANOVA. A p-value of ≤0.05 was considered significant.

## Results

### Neutrophils account for the majority of leukocytes treated by ECP

**Fig 1:**
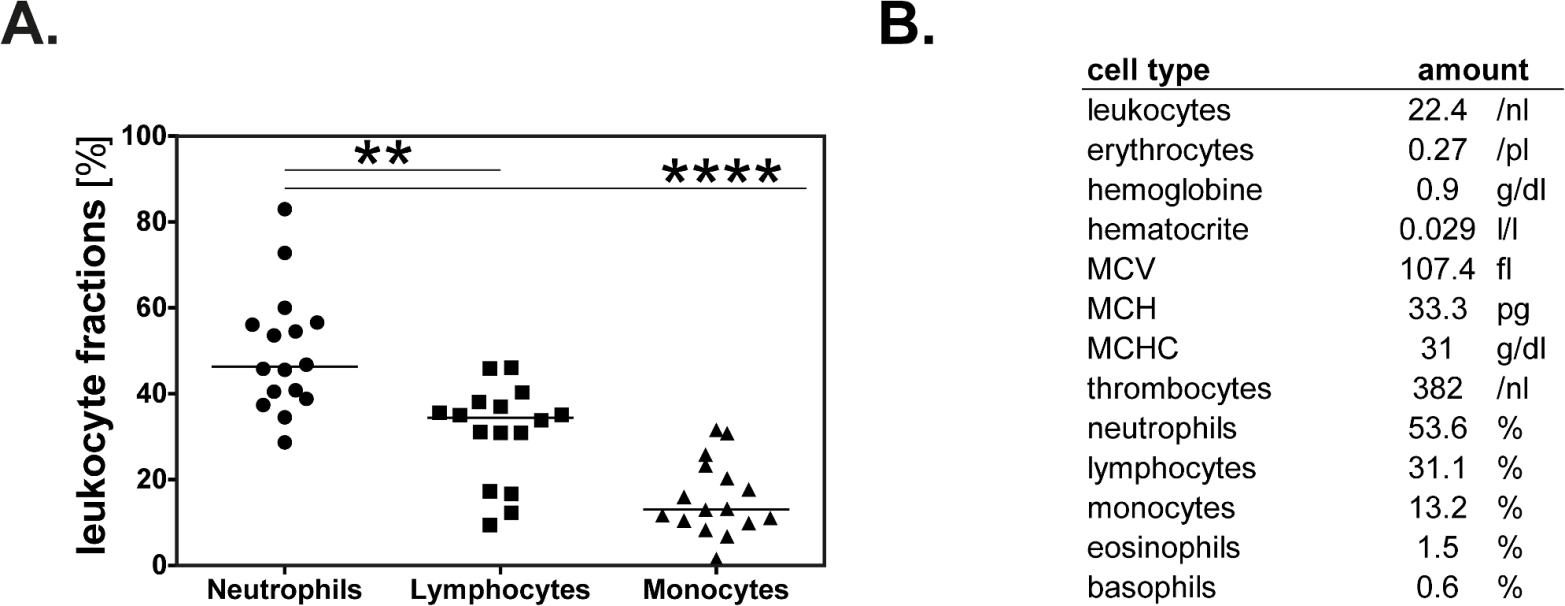
Neutrophils account for the majority of cells treated during ECP. (A) Percentages of neutrophils, lymphocytes and monocytes of total leukocytes in buffy coats of 16 patients with chronic GVHD (cGVHD) taken from Therakos UVAR XTS ECP- device immediately after chemoirradiation prior to reinfusion. Lines show means; scatters represent individual patients. **p≤0.01, ****p≤0.0001. (B) Representative composition of a buffy coat of one patient with cGVHD.

To date, numerous studies on the mechanisms of ECP focused on mononuclear cells. However, analysis of buffy coats from patients with chronic GVHD obtained during ECP showed that the majority of the treated cells belong to the neutrophil fraction. As shown in Fig 1A and 1B, the main leukocyte population found in the buffy coat prior to irradiation was neutrophil granulocytes. In 11/16 patients, neutrophils represent the largest fraction of leukocytes in the buffy coat (between 45.6–83% of the leukocyte fraction, mean 55.65%). In the 5 remaining patients, lymphocytes contributed the majority of treated cells. The neutrophil fraction in these patients varied between 28.5–40.8% of the leukocyte fraction (mean 36.67%) i.e. still accounting for a large fraction of treated cells. The treated lymphocyte fraction showed a range of 9.4–46.1% and a mean of 30.97% and the monocyte fraction ranged from 1.5%-31.6% (mean 15.72%). In summary, the neutrophil fraction is significantly greater than the lymphocyte fraction (p≤0.01) and monocyte fraction (p≤0.0001) treated during ECP.

### *In vitro* treatment of neutrophils with 8-MOP and UVA induces apoptosis

**Fig 2:**
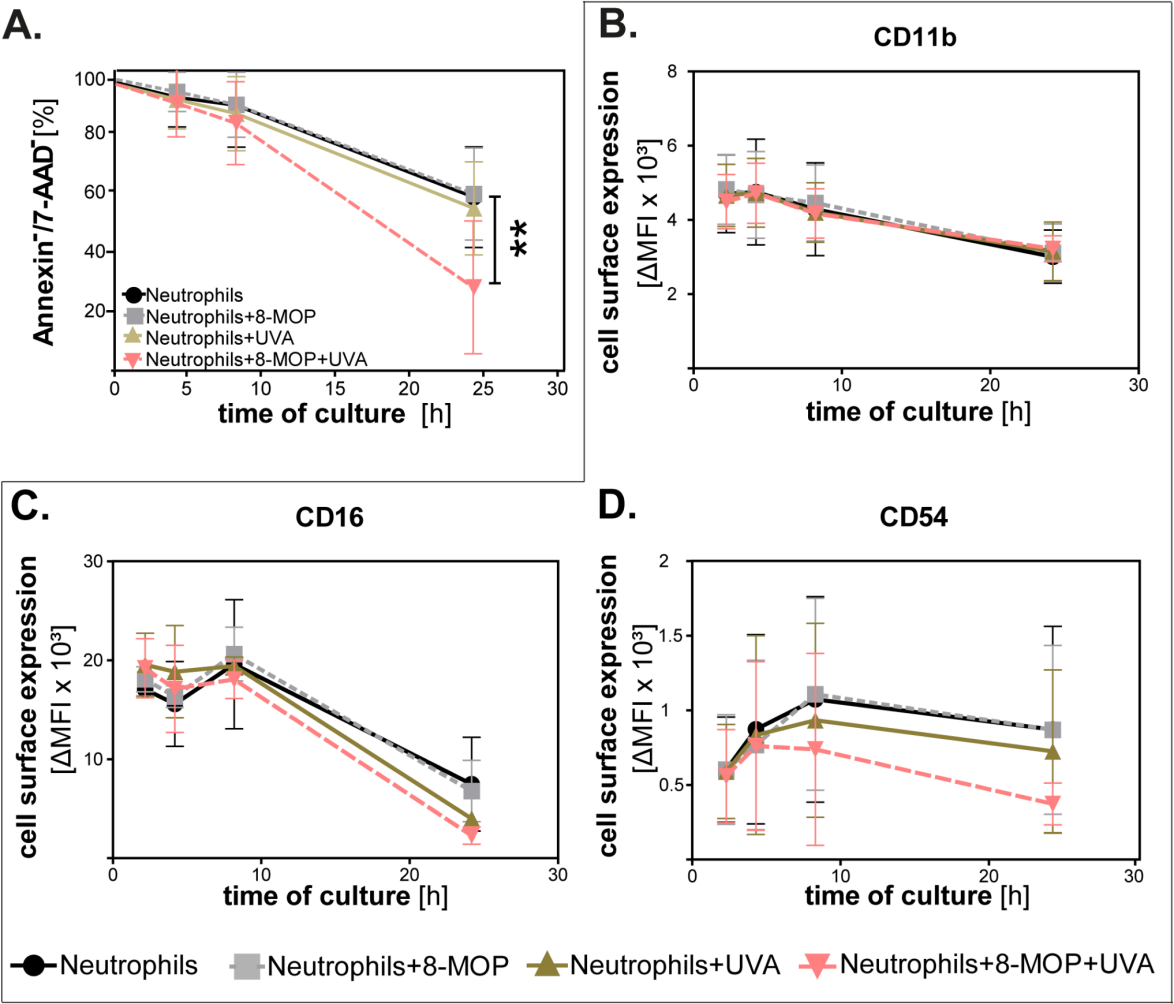
**(A)** *In vitro* treatment of healthy donor neutrophils with 8-methoxypsoralen and UVA induces apoptosis and decreases activation markers. Neutrophils, isolated from blood of healthy donors, were treated with 8-MOP and UVA light and cultured for 24h. Control groups were untreated neutrophils, neutrophils treated with 8-MOP and neutrophils treated with UVA light. Percentage of non-apoptotic cells was determined by the % of Annexin V and 7-AAD double-negative cells via flow cytometry 0, 4, 8 and 24h after irradiation (n = 4). Curves show mean ± standard deviation (SD). ** p≤0.01. **(B-D)** Viable (= Annexin^-^ / 7-AAD^-^) neutrophils of the same groups (n = 4) were stained for activation markers CD11b, CD16 and CD54 at 2, 4, 8 and 24h after irradiation and protein expression was measured by flow cytometry after gating on viable cells. Curves show mean of Δ median of MFI ± SD. MFI = mean fluorescence intensity, Δ median of MFI = MFI of antibody-MFI of isotype shown.

To explore the effects of ECP on neutrophil granulocytes treated during ECP, we first performed *in vitro* experiments with blood samples from healthy donors. *In vitro*, ECP-treatment led to a significant induction of apoptosis in neutrophils 24h after treatment. As shown in Fig 2A, the highest rate of apoptosis was detectable in neutrophils treated with 8-MOP and UVA-light as compared to untreated neutrophils, neutrophils treated with 8-MOP only and neutrophils irradiated with UVA only (p<0.01). At earlier time points (0h, 4h and 8h after treatment), no significant differences between the four groups were detectable. These data suggest that ECP does not induce apoptosis in neutrophils immediately and directly, but rather accelerates spontaneous apoptosis over time.

### *In vitro* treatment with 8-MOP and UVA causes phenotypic changes in neutrophil granulocytes

To detect whether neutrophils showed phenotypic changes after treatment with 8-MOP and UVA, we studied the surface expression of neutrophil markers. At early time points, no phenotypic changes were detectable in treated neutrophils as compared to controls. However, 24h after treatment with 8-MOP and UVA a statistically significant decrease in the expression of the activation markers CD16, CD54 and CD64 was detectable on neutrophils treated with 8-MOP and UVA as compared to control groups (data not shown). CD11b, CD18 and CD32 expression was unaffected by treatment. To exclude that the phenotypic changes observed were solely caused by apoptosis, phenotypic analyses were restricted to Annexin V/7-AAD double-negative, viable neutrophils. As shown in Fig 2B–2D, a marked decrease of CD16 and CD54 expression was still detectable in viable neutrophils treated with 8-MOP and UVA as compared to control groups, while there was no difference detectable for CD11b. Taken together, a loss of activation markers was found in 8-MOP plus UVA treated cells independent of apoptosis. Also, phenotypic changes induced by ECP *in vitro* were selective since CD16 and CD54 appeared to be more affected than CD11b.

### *In vitro* treatment with 8-MOP and UVA affects multiple neutrophil functions

**Fig 3:**
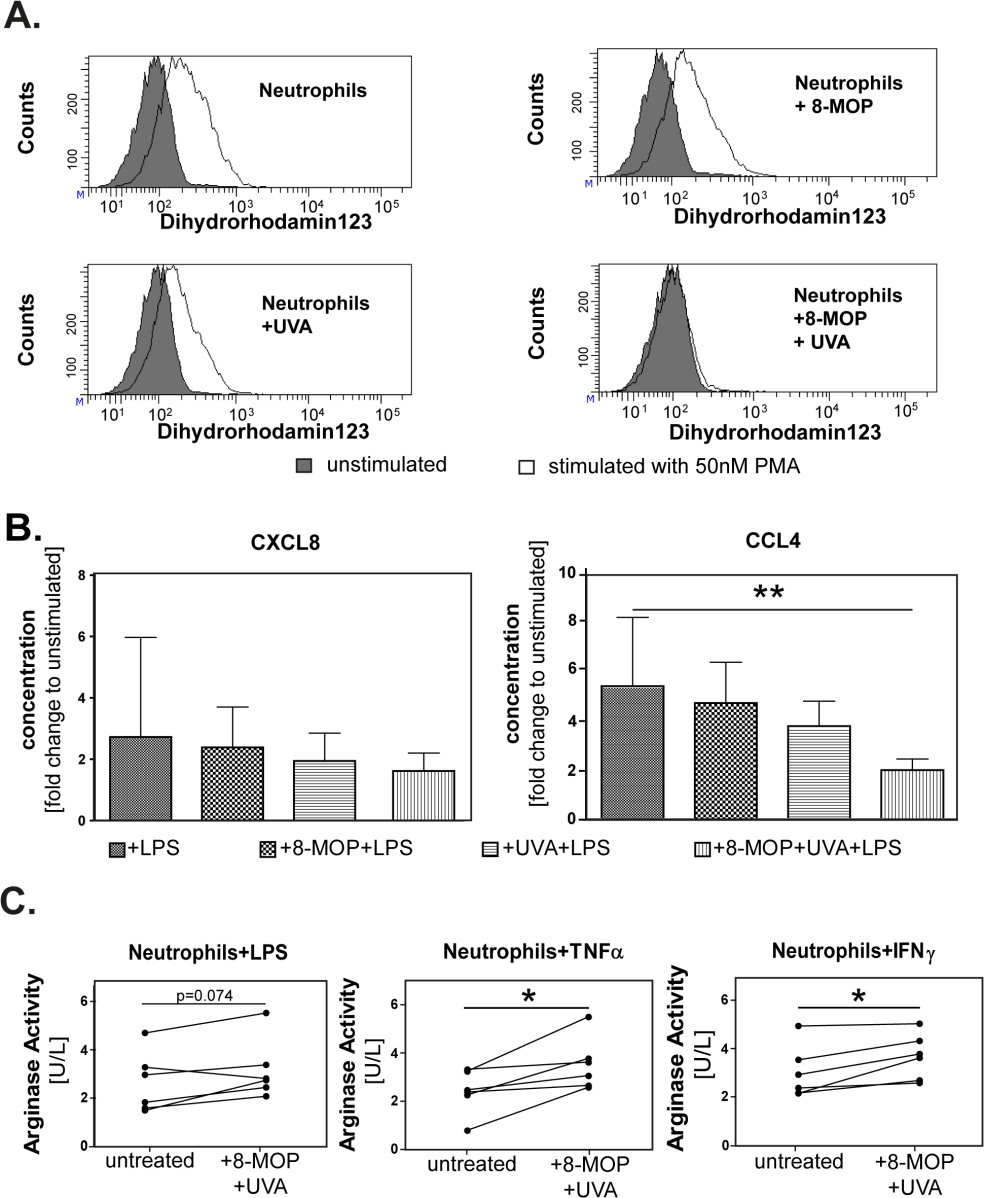
*In vitro* treatment with 8-methoxypsoralen and UVA reduces pro-inflammatory effector functions in healthy donor neutrophils. (A) Neutrophil granulocytes of healthy donors were stimulated with phorbol 12-myristate13-acetate (PMA) for 0, 4, 8 and 24h after treatment with 8-methoxypsoralen and UVA light. Nitric oxide was quantified via flow cytometry by staining with DAF-FM Diacetate after gating on viable cells. Data shown from one representative donor. (B) After 24h of stimulation with LPS, release of CXCL8 and CCL4 into the supernatants was detected by ELISA. Concentrations are shown as x-fold change compared to unstimulated neutrophils. Mean changes of 6 donors ± standard deviation (SD) shown. **p≤0.01. (C) Supernatants of untreated neutrophils and neutrophils treated with 8-MOP and UVA were generated after 24h stimulation with LPS, TNFα and IFNγ (n = 6). Arginase activity was determined enzymatically. Unit definition: 1 unit of arginase converts 1μmol of L-arginine to ornithine and urea per minute at 37°C and pH 9.5. *p = 0.04

Next, we performed functional assays with *in vitro* treated neutrophil granulocytes. Production of reactive nitrogen and oxygen species is a main function of neutrophils. After 24h, neutrophils treated with 8-MOP and UVA showed a significant loss of the ability to release reactive oxygen and nitrogen species in response to PMA-stimulation compared to control groups (untreated neutrophils, neutrophils treated with 8-MOP or UVA light only). Again, apoptosis of neutrophils was not the sole reason for this observation since the Annexin V/ 7-AAD double-negative, non-apoptotic fraction of neutrophils showed a significant decrease of the ability to release RNS compared to the control groups 24h after chemoirradiation (Fig 3A).

To mimic the exposure to bacterial compounds and damage-associated-molecular patterns (DAMPs) released during inflammation and cellular stress [34], neutrophil granulocytes were stimulated with bacterial LPS. Chemoirradiation with 8-MOP and UVA lead to an impaired ability of neutrophil granulocytes to produce chemokines in response to LPS. After 24h of stimulation with LPS neutrophils showed a statistically significant decrease of the ability to release CCL4 (p = 0.0048) and a non-significant decrease of the ability to release CXCL8 (p = 0.5) compared to untreated neutrophils or neutrophils treated with 8-MOP or UVA only (Fig 3B).

Since apoptotic neutrophils are able to release arginase-1, which is an important mechanism to modulate T cell function, we evaluated whether treatment of neutrophils with 8-MOP and UVA leads to an increased extracellular activity of arginase-1. We could show that treatment of neutrophils with 8-MOP and UVA leads to an increase of arginase-1 release into the medium after 24h. This release can be further enhanced by inflammatory cytokines and molecules such as LPS, TNFα or IFNγ. The effect of chemoirradiation was most prominent in neutrophils primed with TNFα and IFNγ (Fig 3C).

### Different apoptosis rates in leukocytes and loss of activation markers of neutrophils after ECP

**Fig 4:**
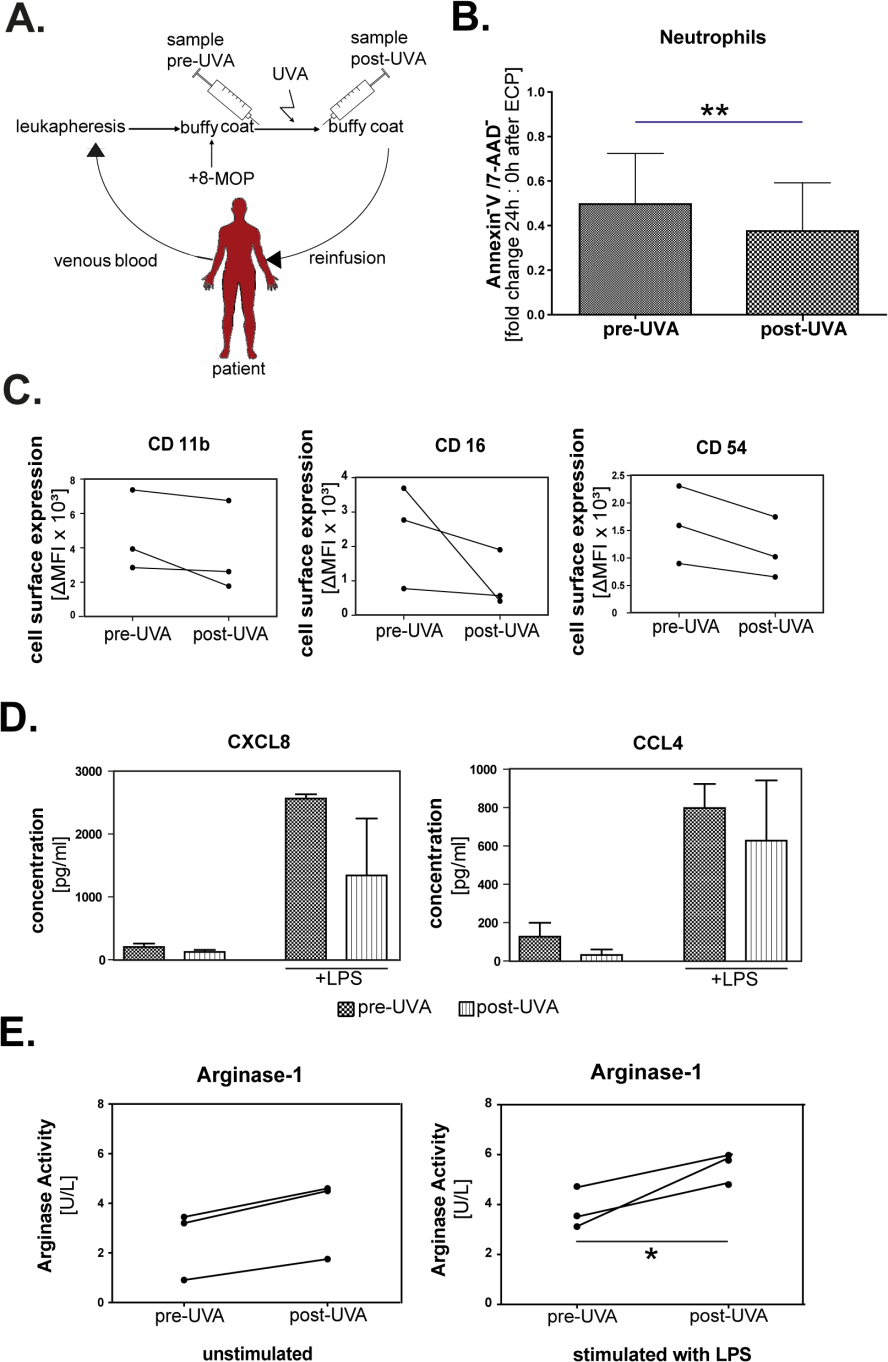
Reduced activation and increased extracellular arginase-1 activity in chemoirradiated neutrophils from buffy coat of cGVHD patients. (A) Neutrophil granulocytes and mononuclear cells substituted with 8-methoxypsoralen (8-MOP) were obtained from the buffy coat of patients with cGVHD treated with a Therakos UVAR XTS device before (pre-UVA) and after (post-UVA) irradiation. (B) Neutrophils of the buffy coat before and after irradiation with UVA (as described in A) were stained with Annexin V and 7-AAD. Fold change of Annexin V^-^/7-AAD^-^double negative (viable) cells determined after 24h of ex vivo culture after treatment normalized to cells assessed directly after treatment (0h). Data show means of 3 patients ± standard deviation (SD). **p≤0.01. (C) Neutrophils from buffy coats of cGVHD patients (n = 3) were stained with antibodies against activation markers CD11b, CD16 and CD54 after 24h in culture. Expression was measured by flow cytometry after gating on viable cells. Data shown as difference of the median of the mean fluorescence intensity (mfi) of the specific antibody and the isotype. (D) Supernatants of neutrophils of the samples described in (A) were generated with and without stimulation with LPS for 24h and concentration of CXCL8 and CCL4 was quantified by ELISA. Mean values ± standard deviation (SD) of 3 patients in pg/ml shown. (E) Arginase activity in the same supernatants as in (D) was determined by enzymatic activity (n = 3). Values shown in U/L. Unit definition: 1 unit of arginase converts 1μmol of L-arginine to ornithine and urea per minute at 37°C and pH 9.5. *p≤0.05

Samples of leukapheresed blood were drawn from the buffy coat bag of 4 patients with chronic GVHD who were undergoing ECP. From each patient, one sample was taken after addition of 8-MOP before irradiation and one sample after irradiation (Fig 4A). Neutrophils and mononuclear leukocytes were stained immediately after isolation and after 24h of culture. In the high density-neutrophil fraction a significant increase of apoptosis rate could be detected during 24h of culture comparing fold change of Annexin V/ 7-AAD-negative cells before (52% non-apoptotic cells) and after (35% non-apoptotic cells) irradiation (Fig 4B). Results within the MNC-fraction (S1 Fig) show the highest apoptosis rate for CD56^+^- cells (average 39.3% non-apoptotic cells after irradiation compared to 86.7% non-apoptotic cells before irradiation), followed by low-density CD66b^+^-granulocytes (49.5% non-apoptotic cells after irradiation vs. 75.7% before irradiation) and CD3^+^-lymphocytes after 24h (58.4% non-apoptotic cells after irradiation vs. 85.6% before irradiation). The lowest apoptosis rate after 24h was detectable in the monocyte fraction (76.6% non-apoptotic cells after irradiation vs. 84.4% before irradiation). Immediately after isolation, no significant differences in apoptosis rates were found between the leukocyte subsets, confirming the data obtained in vitro with cells from healthy donors. Taken together, these data show a differential susceptibility of leukocytes to ECP-induced apoptosis.

In consistence with the data of the *in vitro* treated neutrophils of healthy donors, neutrophils derived from the buffy coat of patients showed a loss of activation markers, most prominently affecting CD16 and CD54 and to a lesser extent also CD11b (Fig 4C). Functionally, we could show a decreased secretion of CXCL8 and CCL4 after chemoirradiation compared to non-chemoirradiated neutrophils when neutrophils were stimulated with LPS for 24h (Fig 4D). In the supernatant of chemoirradiated neutrophils, a significant increase of arginase-1 activity was found after treatment with LPS compared to non-chemoirradiated neutrophils (Fig 4E). Taken together, ECP diminished the capacity of neutrophils to secrete chemokines in response to LPS, yet increased the arginase-activity in the supernatant.

### Analysis of neutrophil granulocytes from peripheral venous blood of patients before and after ECP

**Fig 5:**
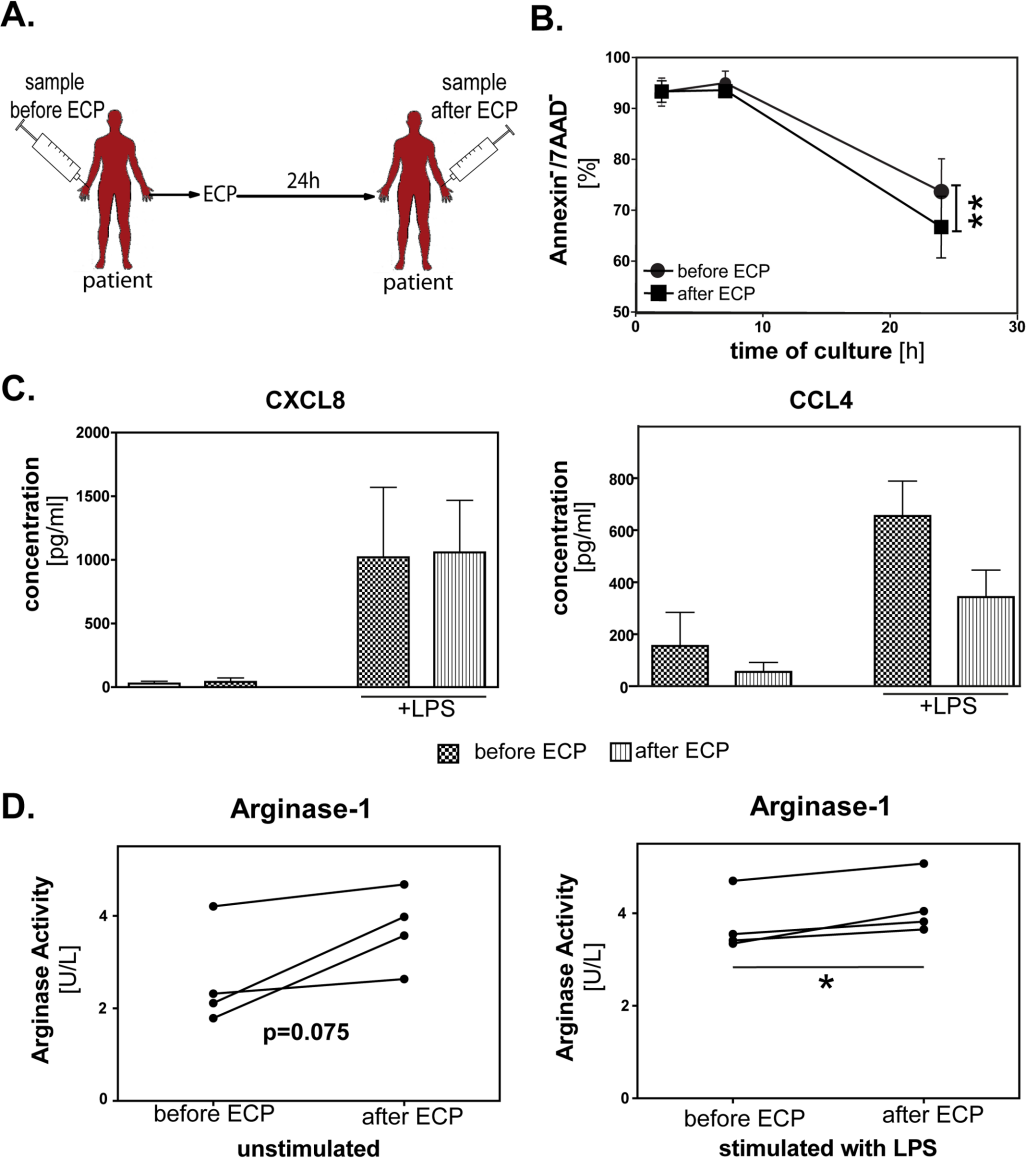
Increased apoptosis and extracellular activity of arginase-1 in neutrophils of cGVHD patients *ex vivo* after ECP. Analysis was performed in neutrophils from peripheral venous blood of patients with cGVHD. (A) Peripheral venous blood samples of patients with cGVHD were taken prior to and 24h after ECP. (B) Apoptosis of neutrophils isolated from these samples was detected immediately after isolation and after 24h of culture via Annexin-V and 7-AAD staining using flow cytometry (n = 4). Data show means of Annexin^-^/7-AAD^-^ (viable) cells ± standard deviation (SD). **p≤0.01. (C) Supernatants were generated of the neutrophils described in (A) +/- LPS for 24h and cytokines CXCL8 and CCL4 were quantified by ELISA. Concentrations are shown in pg/ml as mean of 4 patients ± SD. (D) Arginase-1 activity in these supernatants was determined enzymatically. Arginase activity is shown in U/L. Unit definition: 1 unit of arginase converts 1μmol of L-arginine to ornithine and urea per minute at 37°C and pH 9.5. *p≤0.05.

If alterations in granulocytes are important for treatment of GVHD by ECP, such alterations should be found in neutrophils from venous blood taken by phlebotomy after completion of an ECP cycle. To test this hypothesis, blood samples were taken prior to and 24h after ECP cycles (Fig 5A). Neutrophils isolated from these samples are not directly treated by ECP but represent a mixture of a smaller number of *ex vivo* treated and reinfused leukocytes and a majority of untreated cells. Nevertheless, phenotypic and functional changes resembeling our *in vitro* and *ex vivo* data were found. Neutrophils isolated from blood samples 24h after ECP showed increased apoptosis after *ex vivo* culture compared to neutrophils isolated from samples taken before ECP treatment (p<0.01). At time points before 24h, no significant difference in apoptosis rates between both groups was detectable (Fig 5B). Furthermore, neutrophils isolated from samples taken after ECP show a decrease of their ability to release CCL4, but not CXCL8 (Fig 5C) and an increase of arginase-1 release (p<0.05) after LPS-stimulation (Fig 5D). Neutrophil granulocytes isolated from blood of cGVHD patients 24h after ECP are still capable of lymphocyte suppression (S2A Fig). The suppression is highest at a ratio of 2.5 MNC to 1 neutrophil (90% suppression) and lowest at a ratio of 20 MNC to 1 neutrophil (20% suppression) (S2B Fig). Neutrophils from blood of cGVHD patients after ECP are capable of suppressing both autologous and heterologous lymphocytes.

## Discussion

While the clinical efficacy of ECP in the treatment of GVHD is well established, the immunomodulatory mechanisms of ECP are still not completely understood [18, 35]. Very recent data obtained in a murine model of acute GVHD suggest an important role for neutrophils in the inflammatory processes of this disease [36].

In acute GVHD neutrophils can accumulate in peri-intestinal tissues due to bacterial leakage after conditioning and increase the severity of acute GVHD via ROS production. The role of neutrophils in the chronic type of GVHD has not been assessed in detail. Since neutrophils cleave chemokines, can produce reactive oxygen and nitrogen species and secrete Arginase-1, they are capable of influencing T cell activation and have thereby the potential to contribute to the pathogenesis and maintenance of chronic GVHD as well. Neglected so far, neutrophil granulocytes were studied here as a leukocyte population affected by ECP treatment. We could show that neutrophils are the major leukocyte population treated during ECP. To assess the effects of ECP on neutrophil granulocytes, we established a model that simulates the effects of ECP *in vitro*. Blood samples of healthy donors treated *in vitro* with 8-MOP and UVA were compared to samples of patients with cGVHD from the buffy coat in the ECP-device after apheresis as well as to blood samples of patients with cGVHD before and after ECP. Since our *in vitro* model causes similar functional and phenotypic changes as found on neutrophils from ECP-treated patients analyzed *ex vivo*, our model appears to be highly representative of the events taking place in patients.

Both- neutrophils as well as the therapeutic application of apoptotic cells- can modulate immunopathology in murine models of acute GVHD [37]. In our study, ECP leads to the induction of apoptosis in a large fraction of neutrophil granulocytes and induces a decrease in effector functions in the remaining viable neutrophil fraction of patients with cGVHD. Markedly, in *in vitro* treated neutrophils, as well as neutrophils treated in patients during ECP, there are no immediately visible effects of ECP or effects within the next hours. Effects can only be observed after more than 8h and are statistically significant after 24h. This is probably due to 8-MOP intercalating with DNA double strands in the neutrophils when irradiated with UVA and inducing apoptosis slowly by interference with DNA- replication and inability to produce vital proteins. NK-cells showed the highest susceptibility to treatment with 8-MOP and UVA. Monocytes appeared to be least susceptible. Induction of apoptosis in monocytes has been investigated in several previous studies. Setterblad et al. showed, that ECP treatment sensitized monocytes to spontaneous and HLA-DR mediated cell death [38]. They reported an increase of apoptotic monocytes with a median of 70% after 24h. The lower rate of apoptotic monocytes found in our study corresponds more to the findings of Hannani et al [39]. In their study, monocytes treated with 8-MOP and UVA slowly underwent apoptosis within 6 days with only 20% of the cells showing signs of apoptosis after 24h, but 80% after 6 days. Compared to other leukocytes, NK-cells are most susceptible to treatment with ECP. NK-cells are known to mediate graft-versus-leukemia effects, but their role in ECP treatment has not been analyzed so far. Because NK-cells show the highest susceptibility towards photochemical treatment with 8-MOP and UVA, they could account for an integral part of the apoptotic leukocyte pool induced by ECP. In addition, cytokines produced by NK-cells account for an important mechanism during T cell mediated immune responses. These observations have to be further analyzed in future experiments.

It has been reported that ECP induces rapid maturation of monocytes to dendritic cells [40, 41] and increased apoptosis of alloreactive T lymphocytes. However, direct effects by induction of apoptosis in alloreactive T lymphocytes affect only a minority of alloreactive T cells, since only 5–10% of all leukocytes [20, 21] are exposed to chemoirradiation *ex vivo* of which only a small fraction consists of alloreactive cells. Indirect mechanisms like modulation of dendritic cells and monocytes [42] are alternative mechanisms to explain the immunomodulatory effects caused by ECP. Dendritic cells which are co-incubated with apoptotic T-lymphocytes have been shown to acquire a tolerogenic phenotype and to induce Tregs [43]. Our data suggest, that neutrophil granulocytes might also contribute to the anti-inflammatory properties of ECP. Because of the large number of treated cells, neutrophil granulocytes that are treated by ECP can contribute significantly to the large pool of apoptotic cells, which can lead to the induction of indirect tolerogenic effects on antigen-presenting cells. The results we show also indicate, that ECP decreases the inflammatory activity of neutrophils and induces arginase-1 release from apoptotic neutrophils. Recently, it was shown in a murine model, that neutrophil-derived ROS contributed to the severity of GVHD [36]. Therefore, a decreased ability to release respiratory oxygen and nitrogen species-found in our *in vitro* and *ex vivo experiments*- might lead to reduced tissue damage. Further functional studies showed a reduced secretion of the pro-inflammatory cytokines CXCL8 and CCL4 by neutrophil granulocytes after ECP treatment. Since accumulation of CXCL8 is a feature of steroid-refractory GVHD [44] and CCL4 contributes to accumulation of alloreactive T cells in GVHD, a reduction of CXCL8 and CCL4 secretion from neutrophils might also contribute to the efficacy of ECP. Comparative analysis of blood samples drawn from peripheral veins of patients before and after ECP contain a mix of a small number of ECP-treated and a larger number of non-treated leukocytes. Nevertheless analysis of apoptosis rate and function of neutrophil granulocytes isolated from these samples confirm the findings of our in vitro studies. Based on these findings, it can be concluded that during ECP-treatment a large number of so-called “pre-apoptotic” neutrophils is infused into the patients, which undergo apoptosis in treated patients within the following hours. These pre-apoptotic neutrophils are capable of suppression of T cells according to ex vivo assays. In chronic GVHD defects in central and peripheral tolerance mechanisms can lead to chronic stimulation of CD4^+^ T cells, which in turn activates macrophages, tissue fibroblasts and B cells [45]. Macrophages can further enhance fibroblast activation. Fibrosis by tissue fibroblasts and autoreactive B cells leads to the typical clinical symptoms of chronic GVHD. Suppression of autoreactive T cells might be able to attenuate the activation of macrophages and fibroblasts. The apoptotic neutrophils release large amounts of arginase-1. Arginase-1 is known as one of two key enzymes that can metabolize L-arginine. By doing so, it can modulate the available amounts of extracellular L-arginine. L-arginine is essential for lymphocyte proliferation and IL-2 utilization [46]. L-arginine starvation leads to impairment of T lymphocyte function by triggering internalization of the CD3 zeta chain and impairing cell cycle progression by interfering with the cyclin D3 and cdk4 pathway [47]. Arginase-1 cannot only deplete L-arginine, but also leads to production of immunomodulatory ornithine and polyamines, which can also inhibit maturation of T-lymphocytes [48]. Although Arginase-1 secretion is one of the major T cell suppressive mechanisms of neutrophils, the molecular mechanism of T cell suppression in our setting still needs to be elucidated in the future. Previous studies in mice showed, that a population of murine MDSC inhibit GVHD via an arginase-1- dependent mechanism [49]. Merlin et al. demonstrated, that ECP-treatment of patients who suffered from GVHD, lead to an increase of arginase-1 activity and expression in the blood of these patients and that by this mechanism important inflammatory cytokines could be regulated [50]. However, this effect was attributed to the mononuclear cell fraction treated by ECP. Based on our data, we hypothesize that increasing serum levels of arginase-1 found during the course of ECP treatment are derived from neutrophils, rather than from mononuclear leukocytes. *In vitro*, ECP-induced arginase-1 activity was further increased by IFNγ. This might be of critical importance since IFNγ contributes to the pathogenesis of GVHD and is found on increased levels in patients with GVHD [51]. This hypothesis would also be in accordance with the findings of Rieber et al. who showed an increase of arginase-1 in the serum of patients and the supernatants of low-density granulocytes [30]. Since these low-density granulocytes account only for a very small number of total leukocytes (less than 1% in healthy individuals), whereas conventional granulocytes analyzed in our study are the major leukocyte subset affected by ECP, it is very likely that arginine consumption in cGVHD patients is affected by the mechanisms described in this report.

In sum, we have described anti-inflammatory modulation and induction of apoptosis of neutrophil granulocytes by ECP and propose that these mechanisms contribute to the clinical efficacy of ECP against cGVHD in patients.

## Conclusion

The present study is the first to systematically address the effects of extracorporeal photopheresis on neutrophil granulocytes in the treatment of cGVHD. Neutrophils do not just account for the largest cell fraction treated during ECP, but can potentially modulate T cell proliferation in several ways. Our results describe two potential new mechanisms of action of ECP. Firstly, enhanced apoptosis of neutrophils taking place *in vivo* leads to a decrease of inflammatory activity and tissue damage by reduced secretion of pro-inflammatory cytokines and respiratory oxygen and nitrogen species. Secondly, chemoirradiated neutrophils directly release immunomodulatory molecules such as arginase-1. Additional indirect effects of apoptotic granulocytes on antigen-presenting cells are to be expected and need to be investigated further in future projects. The functional consequences of these mechanisms and modulation of immunoreactivity by photochemically treated neutrophils depict a novel hypothesis to the mechanisms of action of ECP.

## Supporting Information

S1 Fig Comparative analysis of apoptosis in leukocytes after ECP. Leukocytes from cGVHD patients taken from buffy coat before and after chemoirradiation were stained for Annexin-/7-AAD- double negative cells immediately after ECP and after 24h. *p≤0.05, **p≤0.01
(TIF)

S2 Fig Suppression of lymphocytes by neutrophils *ex vivo* after ECP. Neutrophils isolated from 3 patients with chronic GVHD were added to autologous (upper row) or heterologous (lower row) monocyte-depleted mononuclear cell fractions in different ratios after labelling of mononuclear cells with cell proliferation dye efluor 450. T-cells were stimulated with plate-bound antibodies against CD3 and CD28. Proliferation was measured via flow cytometry after 5 days of proliferation and gating on the lymphocyte fraction. (A) representative data of one donor showing proliferation of lymphocytes. (B) Mean of suppression of 3 donors ± standard deviation.
(TIF)

S1 Table List of antibodies used for flow cytometric analysis of activation markers. (TIF)
