# Supplementary figures and images for: Modulation and Apoptosis of Neutrophil Granulocytes by Extracorporeal Photopheresis in the Treatment of Chronic Graft-Versus-Host Disease
Source: PLoS One. 2015 Aug 4;10(8):e0134518. doi: 10.1371/journal.pone.0134518 (PMC4524718; doi:10.1371/journal.pone.0134518)

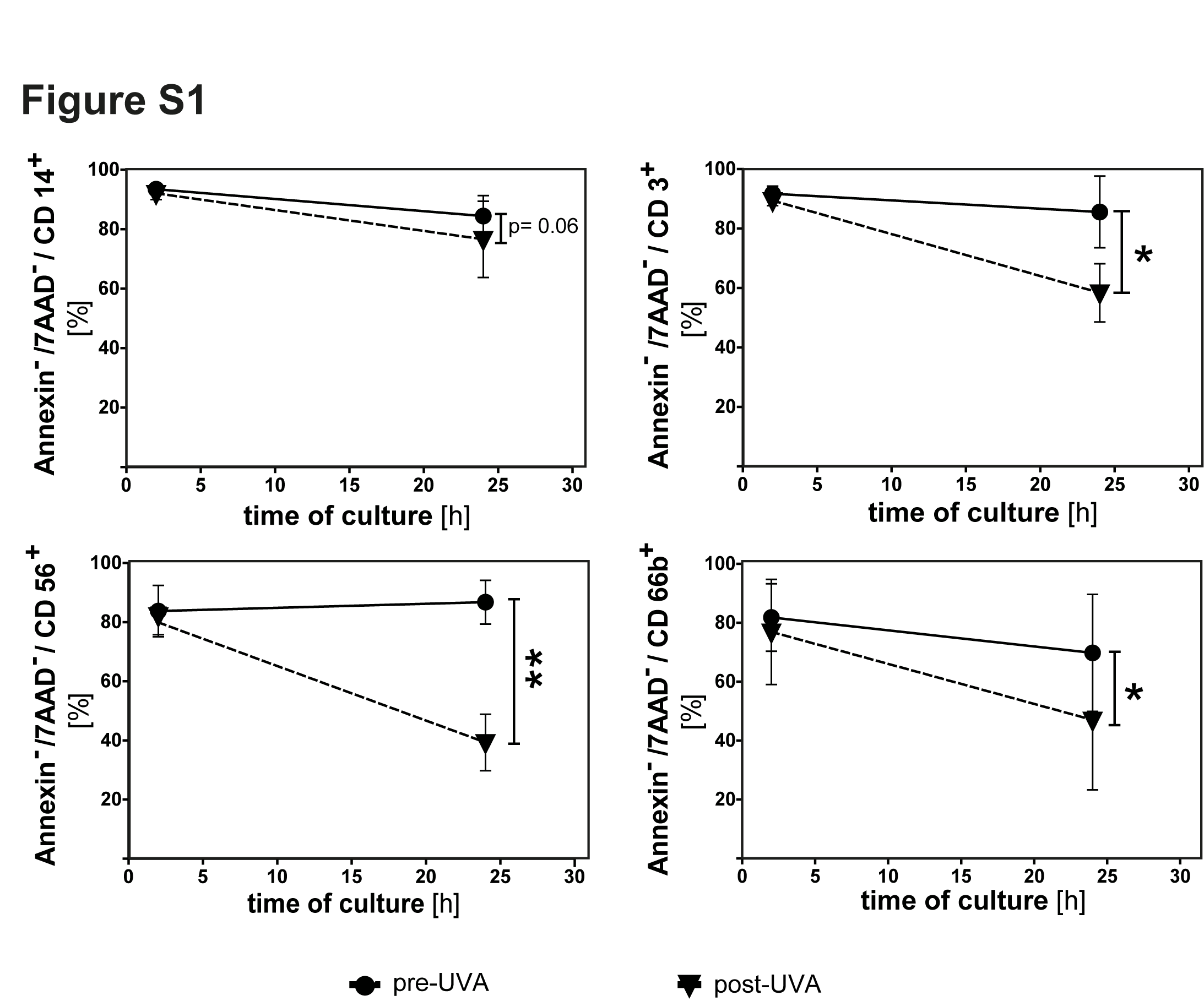

Supplement: S1 Fig — Leukocytes from cGVHD patients taken from buffy coat before and after chemoirradiation were stained for Annexin-/7-AAD- double negative cells immediately after ECP and after 24h. *p≤0.05, **p≤0.01 (TIF) [file pone.0134518.s001.tif]

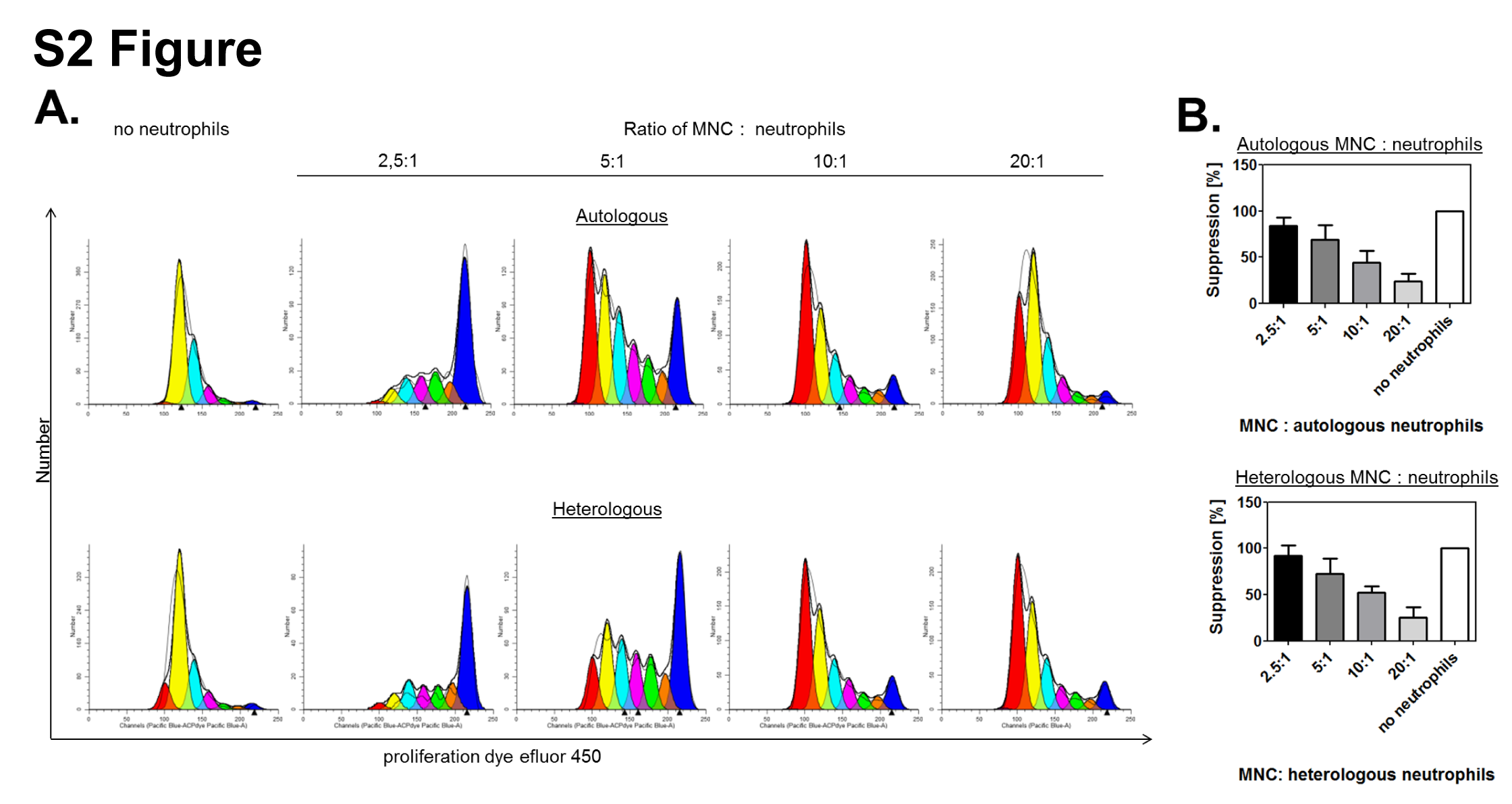

Supplement: S2 Fig — Neutrophils isolated from 3 patients with chronic GVHD were added to autologous (upper row) or heterologous (lower row) monocyte-depleted mononuclear cell fractions in different ratios after labelling of mononuclear cells with cell proliferation dye efluor 450. T-cells were stimulated with plate-bound antibodies against CD3 and CD28. Proliferation was measured via flow cytometry after 5 days of proliferation and gating on the lymphocyte fraction. (A) representative data of one donor showing proliferation of lymphocytes. (B) Mean of suppression of 3 donors ± standard deviation. (TIF) [file pone.0134518.s002.tif]

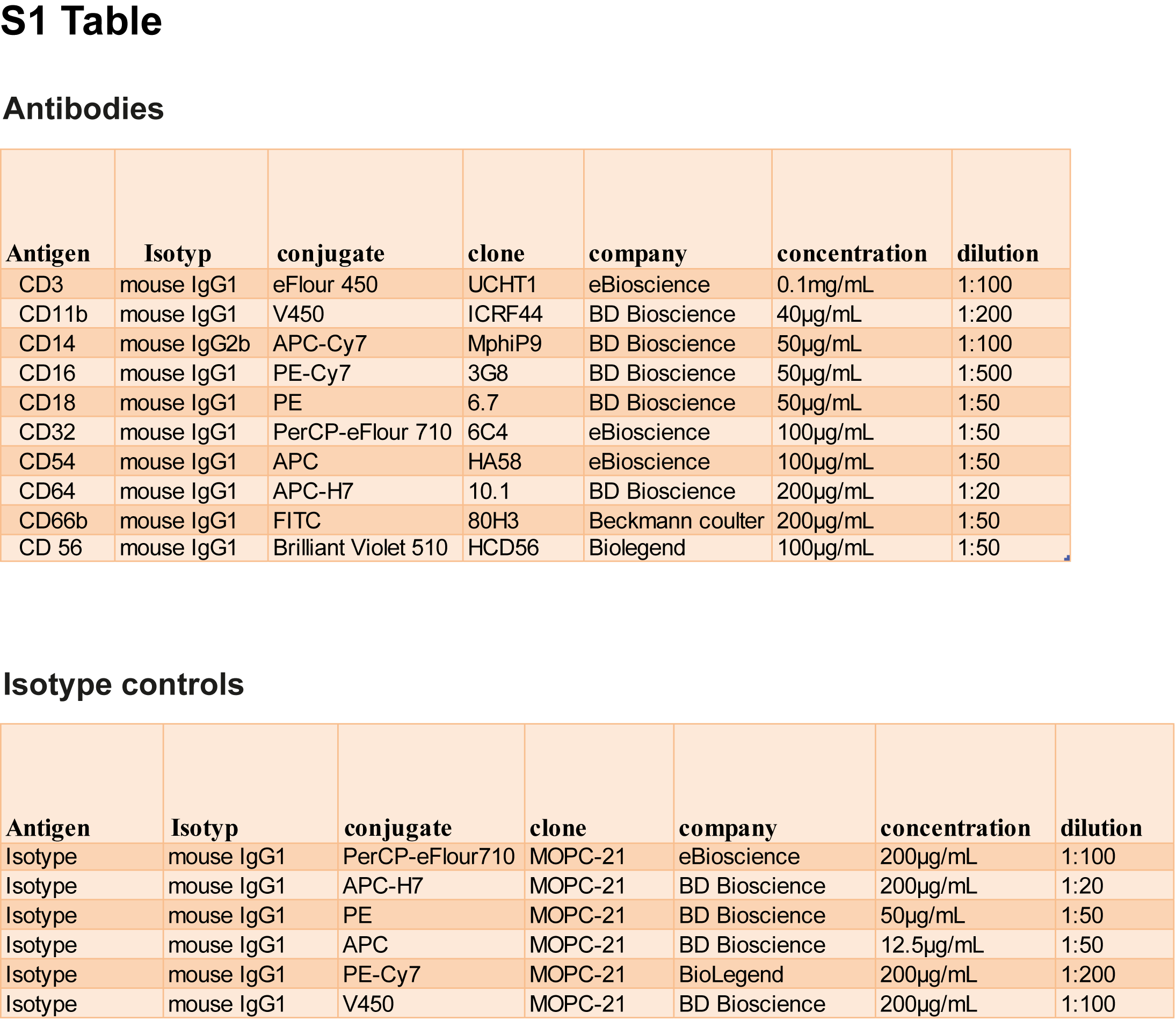

Supplement: S1 Table — (TIF) [file pone.0134518.s003.tif]
